# Stereogram of the Living Heart, Lung, and Adjacent Structures

**DOI:** 10.3390/tomography8020068

**Published:** 2022-03-17

**Authors:** Yu Izawa, Tatsuya Nishii, Shumpei Mori

**Affiliations:** 1 Division of Cardiovascular Medicine, Department of Internal Medicine, Kobe University Graduate School of Medicine, Kobe, Hyogo 650-0017, Japan; yizawa@people.kobe-u.ac.jp; 2 Department of Radiology, National Cerebral and Cardiovascular Center, Suita, Osaka 564-8565, Japan; ttsynishii@ncvc.go.jp; 3 UCLA Cardiac Arrhythmia Center, UCLA Health System, David Geffen School of Medicine at UCLA, Los Angeles, CA 90095, USA

**Keywords:** anaglyph, anatomy, computed tomography, heart, stereogram

## Abstract

Innovations in invasive cardiovascular diagnostics and therapeutics, not only limited to transcatheter approaches but also involving surgical approaches, are based on a precise appreciation of the three-dimensional living heart anatomy. Rapid advancements in three-dimensional cardiovascular imaging technologies in the 21st century have supported such innovations through the periprocedural assessment of the clinical anatomy of the living heart. However, even if high-resolution volume-rendered images are reconstructed, they cannot provide appropriate depth perception when displayed and shared on a two-dimensional display, which is widely used in clinical settings. Currently, images reconstructed from clinical datasets can visualize fine details of the cardiovascular anatomy. Therefore, this is an optimal time for cardiologists and cardiac surgeons to revisit the classic technology, stereopsis, and obtain bonus information from carefully reconstructed clinical images. Using anaglyphs or cross/uncross-fusion of paired images, striking depth perception can be readily obtained without the need for expensive equipment. This conventional technique, when applied to high-resolution volume-rendered images, may help in obtaining appropriate diagnostics, choosing optimal therapeutics, securing procedural success, and preventing complications. Furthermore, it can be used for anatomical education. In this review, we demonstrate multiple stereoscopic images reconstructed from cardiac computed tomographic datasets and discuss their clinical and educational implications.

## 1. Introduction

A three-dimensional understanding of the heart is essential for cardiovascular diagnostics and therapeutics because the heart is a three-dimensionally complicated organ. Conventionally, however, two-dimensional evaluation of the cardiac anatomy has been the mainstream in the clinical practice of cardiology, including chest radiography, transthoracic echocardiography, and coronary angiography. These two-dimensional images involve either sectional images, such as transthoracic echocardiography images, or projection images, such as chest radiography and coronary angiography images. Therefore, the three-dimensionality of each heart in real time is restored by reconstructing the solid object mentally from multiple serial sectional images or by trying to “project back” a single projection image from the panel toward the tube. However, such experience-based and imagination-driven three-dimensional perceptions cannot always be communicated and recognition errors cannot be avoided. With rapid advancements in the recent decades, three-dimensional cardiovascular imaging has become a part of daily clinical practice, including three-dimensional echocardiography [1,2] and cardiac computed tomography [3,4,5]. However, owing to their two-dimensional display, these three-dimensional images cannot provide real depth perception. Equipment that overcomes this limitation includes three-dimensional printing, three-dimensional projectors/monitors, and virtual reality applications, which enhance real three-dimensional visualization with depth perception [6,7,8,9,10,11]. However, these new methodologies have limitations in terms of user-friendliness, cost performance, and clinical relevance, which prevent them from being widely applied in clinical practice.

Binocular stereopsis based on binocular disparity was first described by Wheatstone in 1838 [12]. To the best of our knowledge, this simple method to obtain real depth perception was applied to the field of cardiology in 1908 by DeWitt, who showed a model of the cardiac conduction system [13]. The usefulness of this conventional method in the field of cardiology has recently regained focus [4,14,15,16]. The convenience of this technique, without the use of expensive equipment, could vitalize the three-dimensional images currently demonstrated on a two-dimensional surface. In this review, we present stereoscopic images of various cardiac diseases and demonstrate their clinical and educational usefulness.

## 2. Concept and Types of Binocular Stereopsis

The concept of binocular stereopsis is that retinal images of the left and right eyes differ because of pupil distance [12]. These two two-dimensional scenes are reproduced as a single image in the visual cortex of the occipital lobe of the brain, recovering the three-dimensional world with depth [17,18]. A set of these two-dimensional images is referred to as a “stereogram,” which has multiple types. Although stereograms can be displayed in various ways, for convenience, we prepared parallel (for cross-eyed viewing) and single-image stereograms (for anaglyphic viewing) for this review. Unlike parallel viewing, cross-eye viewing helps achieve stereopsis, even with larger images. An anaglyph requires special red/cyan glasses. However, unlike parallel-image stereograms, it does not require stereoscopic practice. A disadvantage of anaglyphs is the loss of natural color due to the red/blue tint in vision.

## 3. Preparation of a Stereogram

We reconstructed representative volume-rendered images from multiple patients who underwent contrast-enhanced electrocardiography-gated cardiac computed tomography using a commercially available dual-source computed tomographic scanner (SOMATOM Force, Siemens Healthcare, Forchheim, Germany) and a 256-detector row-computed tomographic scanner (Revolution CT, GE Healthcare, Milwaukee, WI, USA). Informed consent was obtained from all patients before the procedure. All image reconstructions were performed using a commercially available workstation (Ziostation2, version 2.9.8.4; AMIN Co., Ltd., Tokyo, Japan; Ziosoft Inc., Tokyo, Japan). To display stereoscopic images, a pair of volume-rendered images with 10° differences in the horizontal rotation angle was exported and appropriately aligned for the stereoscopic display for cross-eyed viewing. Using the same paired images, anaglyphs were generated using freeware (StereroPhoto Maker Pro, version 6.19). To optimize visualization, a half-color (red/cyan) setting was applied.

## 4. Representative Images

### 4.1. Attitudinal Position of the Living Heart within the Chest

**Figure 1:**
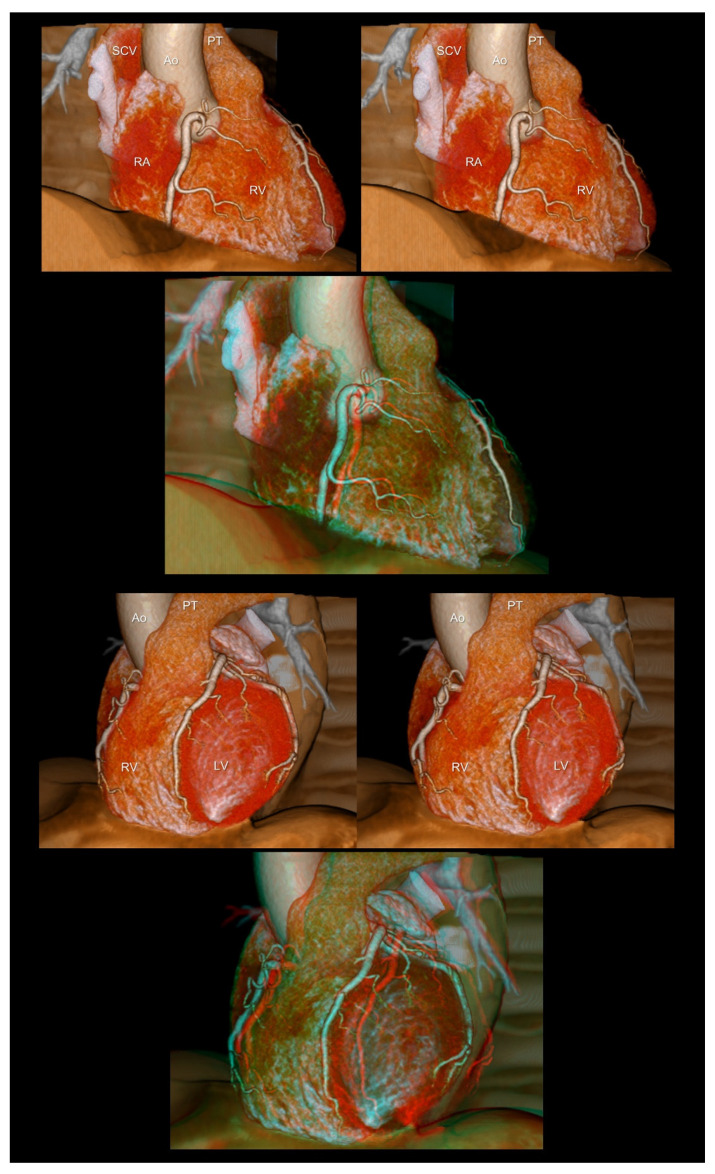
The right (**upper panels**) and left (**lower panels**) anterior oblique views of the heart reconstructed without epicardial adipose tissue. The first and second panels are aligned for cross-eyed viewing. The third panel is the anaglyph. To see the anaglyph, anaglyphic glasses (red/cyan) are required. Ao, ascending aorta; LV, left ventricle; PT, pulmonary trunk; RA, right atrium; RV, right ventricle; SCV, superior caval vein.

An appreciation of the physiological location of the three-dimensional living heart within the chest is fundamental before diving into the in-depth cardiovascular diagnostics, including inspection, palpation, percussion, auscultation, chest radiography, electrocardiography, and echocardiography. A living heart visualized in an attitudinally appropriate fashion can show which structure is superior/inferior, anterior/posterior, and right/left, although many confusing terminologies are used in routine clinical settings [3,19]. In addition to the frontal direction, the right and left anterior oblique directions are important for differentiating the atrium from the ventricle, and the right from the left side of the heart (Figure 1).

### 4.2. Coronary Arteries

**Figure 2:**
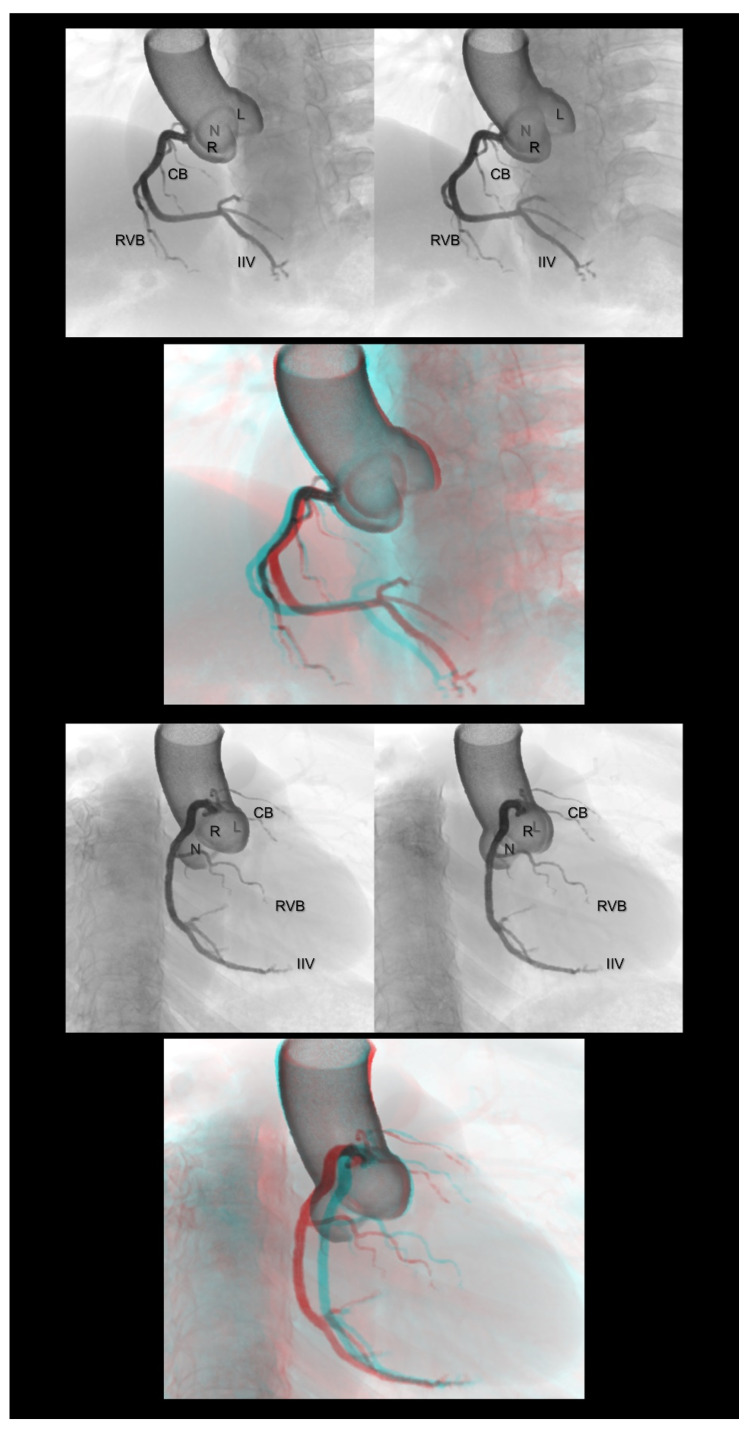
Left anterior oblique cranial view (**upper panels**) and right anterior oblique caudal view (**lower panels**) of the right coronary artery. Three-dimensional understanding of the coronary angiography in relation to each coronary aortic sinus is fundamental before learning the procedure. The first and second panels are aligned for cross-eyed viewing. The third panel is the anaglyph. To see the anaglyph, anaglyphic glasses (red/cyan) are required. CB, conus branch; IIA, inferior interventricular artery; L, left coronary aortic sinus; N, noncoronary aortic sinus; R, right coronary aortic sinus; RVB, right ventricular branch.

**Figure 3:**
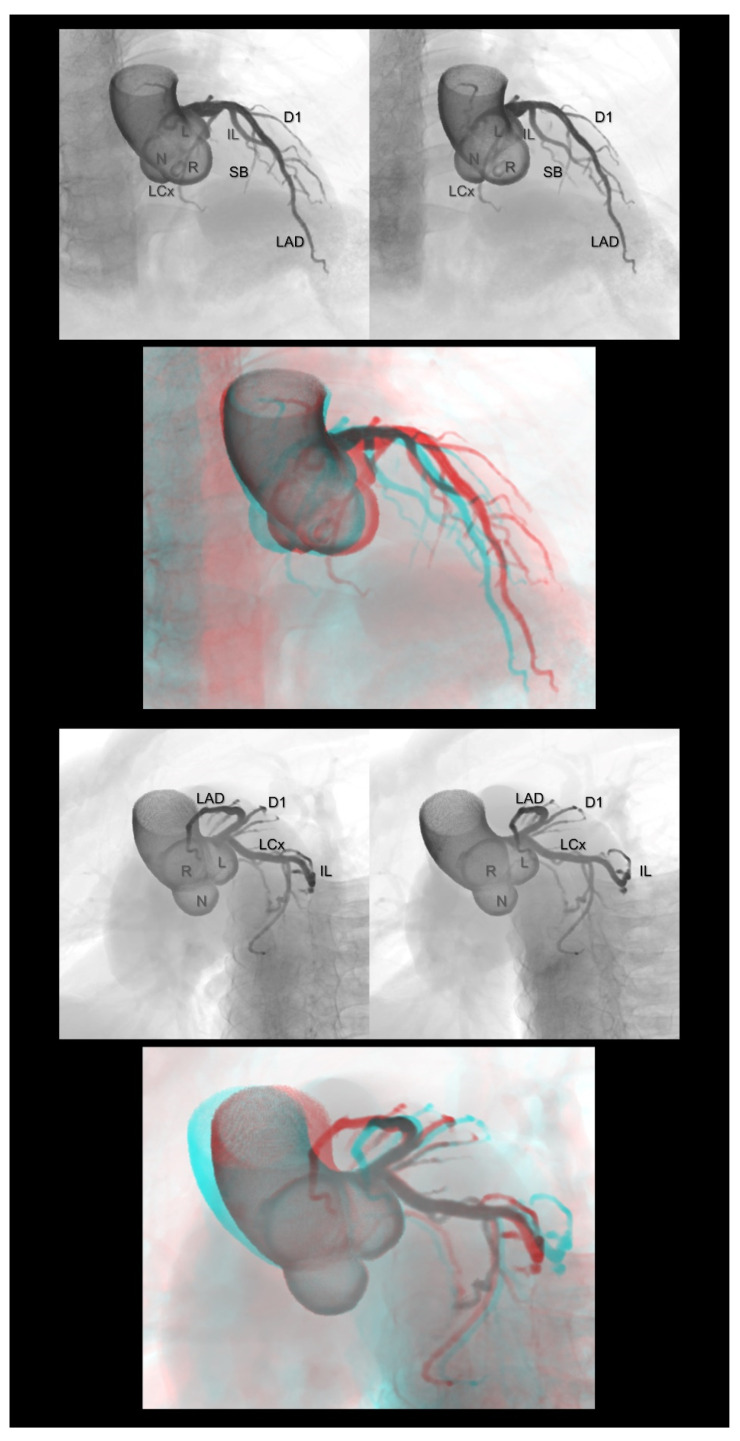
Right anterior oblique cranial view (**upper panels**) and left anterior oblique caudal view or spider view (**lower panels**) of the left coronary artery. The first and second panels are aligned for cross-eyed viewing. The third panel is the anaglyph. To see the anaglyph, anaglyphic glasses (red/cyan) are required. D1, first diagonal branch; IL, inferolateral branch; L, left coronary aortic sinus; LAD, left anterior descending artery; LCx, left circumflex artery; N, noncoronary aortic sinus; R, right coronary aortic sinus; SB, septal branch.

Coronary angiography is a basic procedure used by cardiologists in clinical practice. However, the precise perception of the three-dimensionality of representative coronary angiograms displayed on two-dimensional monitors is difficult for medical students, non-cardiologists, and trainees in cardiology and cardiovascular surgery. Stereograms are simple solutions for easy and precise three-dimensional recognition of angiograms (Figure 2 and Figure 3). For example, particularly for beginners, the left anterior oblique and caudal views of the left coronary artery, also referred to as the spider view, are generally observed as if the peripheral vessels are directed away from the observer. This type of imaginary confusion can be avoided using a stereogram. Using this technique, it is easy to recognize which part of the coronary artery runs toward or away from the observer, or how they can be separated and visualized without foreshortening from each angulation. Such precise three-dimensional recognition is the basis for accurate diagnoses during coronary angiography and eventually supports smooth and safe therapeutics.

### 4.3. Coronary Veins

**Figure 4:**
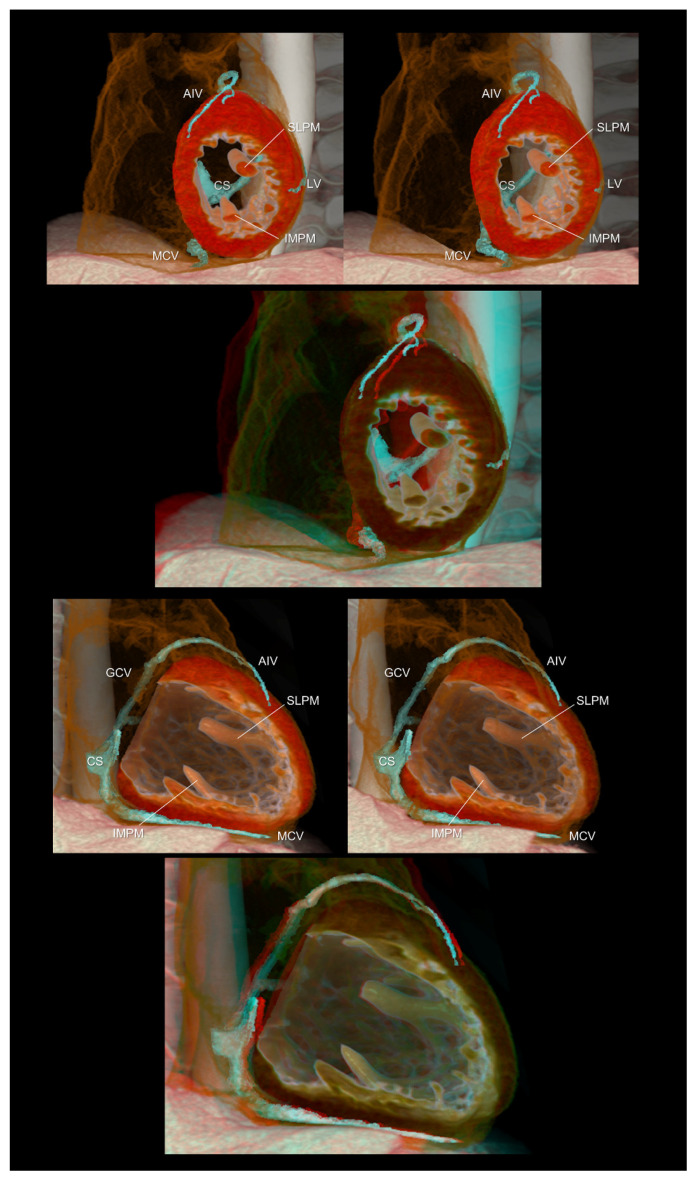
Left ventricular short (**upper panels**) and long (**lower panels**) axis images showing the relationship between the coronary veins and the left ventricular structures. The lateral vein, a candidate for left ventricular lead insertion for cardiac resynchronization therapy, runs in the region between the superolateral and inferomedial papillary muscles. The first and second panels are aligned for cross-eyed viewing. The third panel is the anaglyph. To see the anaglyph, anaglyphic glasses (red/cyan) are required. AIV, anterior interventricular vein; CS, coronary sinus; GCV, great cardiac vein; IMPM, inferomedial papillary muscle; LV, lateral vein; MCV, middle cardiac vein; SLPM, superolateral papillary muscle.

The three-dimensional living anatomy of the coronary venous system is important for cardiac resynchronization therapy [20] and epicardial ablation [21,22]. In both settings, it is important to appreciate its relationship with the surrounding structures because the target should not be the vein itself but the adjacent myocardium [23]. Figure 4 illustrates this structural relationship.

### 4.4. Pulmonary Arteries

**Figure 5:**
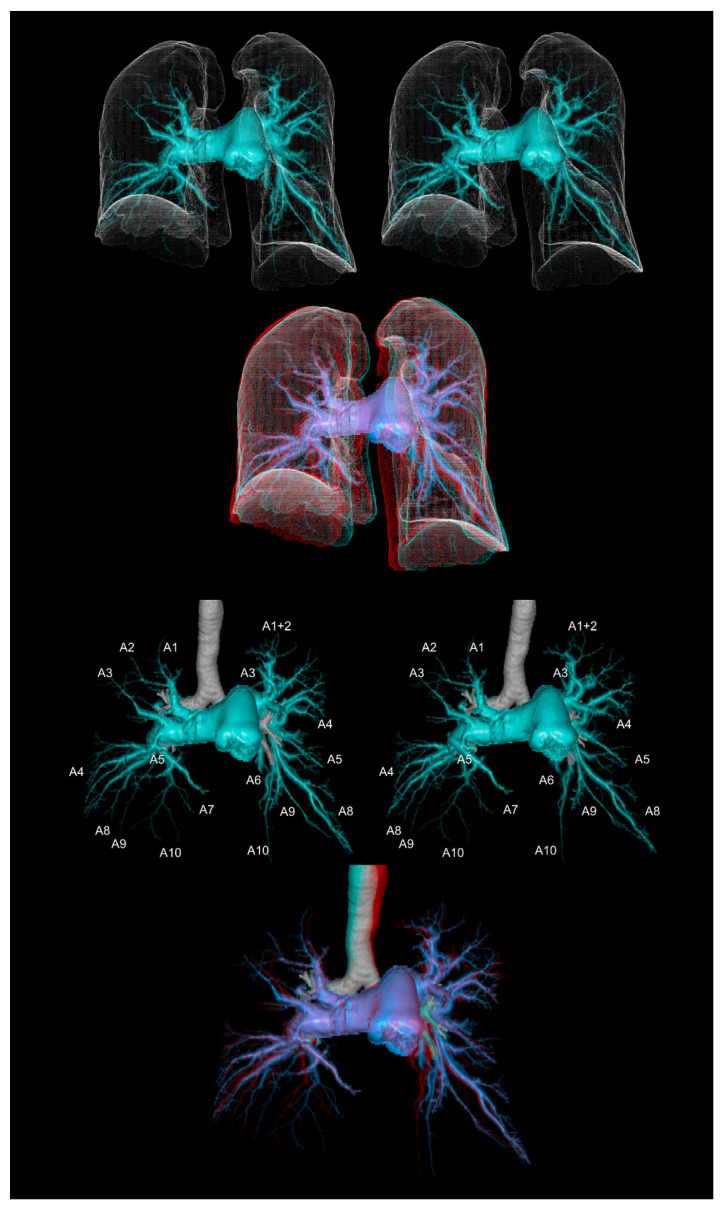
The computed tomographic pulmonary angiography of a patient with chronic thromboembolic pulmonary hypertension. The upper panels show the pulmonary arteries with the lung contours. The lower panels show the pulmonary arteries with the trachea and main bronchi. The first and second panels are aligned for cross-eyed viewing. The third panel is the anaglyph. To see the anaglyph, anaglyphic glasses (red/cyan) are required.

The pulmonary trunk gives off the right and left pulmonary arteries, which then divide into lobar branches before further bifurcation into branches that are distributed to each segment. In contrast with the right pulmonary artery, the left pulmonary artery overrides the left main bronchus. However, the peripheral pulmonary artery/arteriole runs parallel to the bronchus/bronchiole. With the establishment of diagnostics and therapeutics for chronic thromboembolic pulmonary hypertension [24], it is necessary for clinicians to understand the three-dimensional anatomy of the pulmonary artery in relation to the bronchopulmonary segment of the lungs. However, compared with coronary angiography, it is difficult to perceive the three-dimensional relationship of the pulmonary artery from pulmonary angiography displayed on a two-dimensional monitor. In this regard, stereograms provide striking depth perception in relation to the lungs and bronchi (Figure 5).

### 4.5. Valvar Heart Diseases

**Figure 6:**
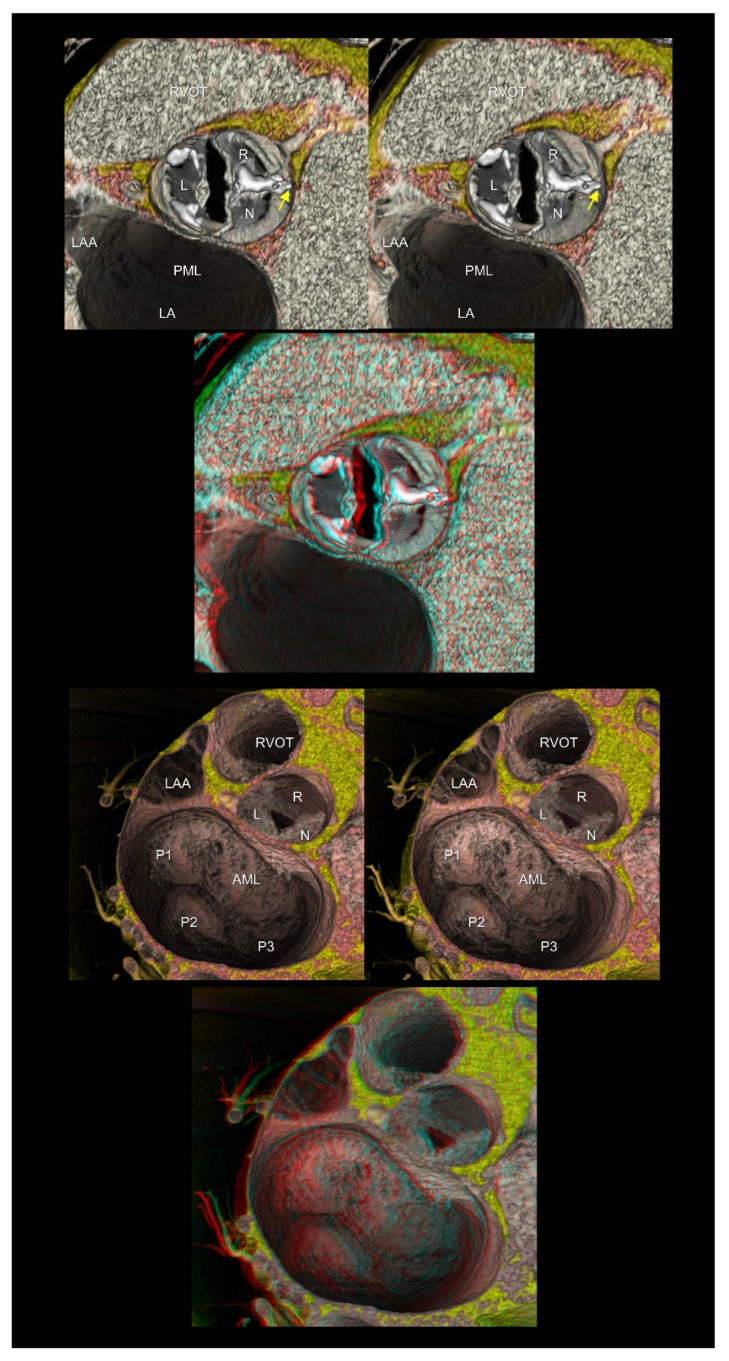
The upper panels show the functionally bileaflet (bicuspid) and trisinuate aortic root. Fusion with the calcified raphe is found between the right and noncoronary aortic leaflets. The apex of the hypoplastic interleaflet triangle between the fused leaflets (yellow arrow) does not reach the plane of the sinutubular junction. The lower panels represent mitral annular dilatation with extensive billowing/prolapse of both mitral leaflets, consistent with Barlow’s disease. The first and second panels are aligned for cross-eyed viewing. The third panel is the anaglyph. To see the anaglyph, anaglyphic glasses (red/cyan) are required. AML, anterior mitral leaflet; L, left coronary aortic sinus; LA, left atrium; LAA, left atrial appendage; N, noncoronary aortic sinus; PML, posterior mitral leaflet; P1, lateral scallop of the PML; P2, middle scallop of the PML; P3, medial scallop of the PML; R, right coronary aortic sinus; RVOT, right ventricular outflow tract.

Transcatheter treatment for valvar heart diseases [25,26] and valve-sparing surgery [27,28] are emerging fields related to valvar heart diseases. As any of the heart valves and their relationship with the surrounding structures are three-dimensionally complex [29], two-dimensional images are not intuitive, occasionally misleading [5], and insufficient for a comprehensive evaluation. In this regard, the preprocedural detailed assessment of valvar morphology using cardiac computed tomography is an attractive option [30,31,32] in cases involving preprocedural computed tomographic evaluation of coronary artery stenosis. For the aortic and mitral valves, a careful reconstruction using the volume-rendering method can demonstrate the detailed three-dimensional anatomy, providing additional depth perception with stereograms, which is relevant to discern the precise etiology and select an optimal therapeutic strategy to restore the appropriate structure and normal function (Figure 6).

### 4.6. Congenital Heart Disease

**Figure 7:**
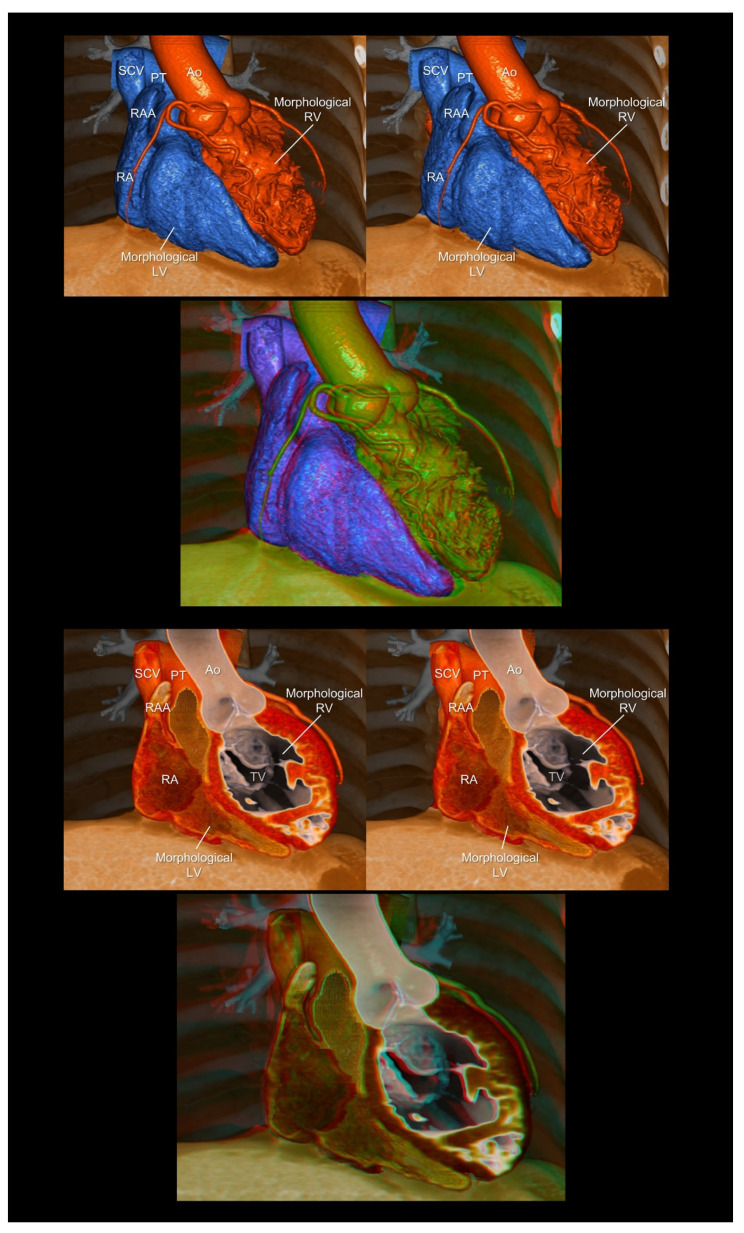
Endocast (**upper panels**) and virtual dissection images (**lower panels**) of the corrected transposition of the great arteries. The first and second panels are aligned for cross-eyed viewing. The third panel is the anaglyph. To see the anaglyph, anaglyphic glasses (red/cyan) are required. Ao, aorta; LV, left ventricle; PT, pulmonary trunk; RA, right atrium; RAA, right atrial appendage; RV, right ventricle; SCV, superior caval vein; TV, tricuspid valve.

**Figure 8:**
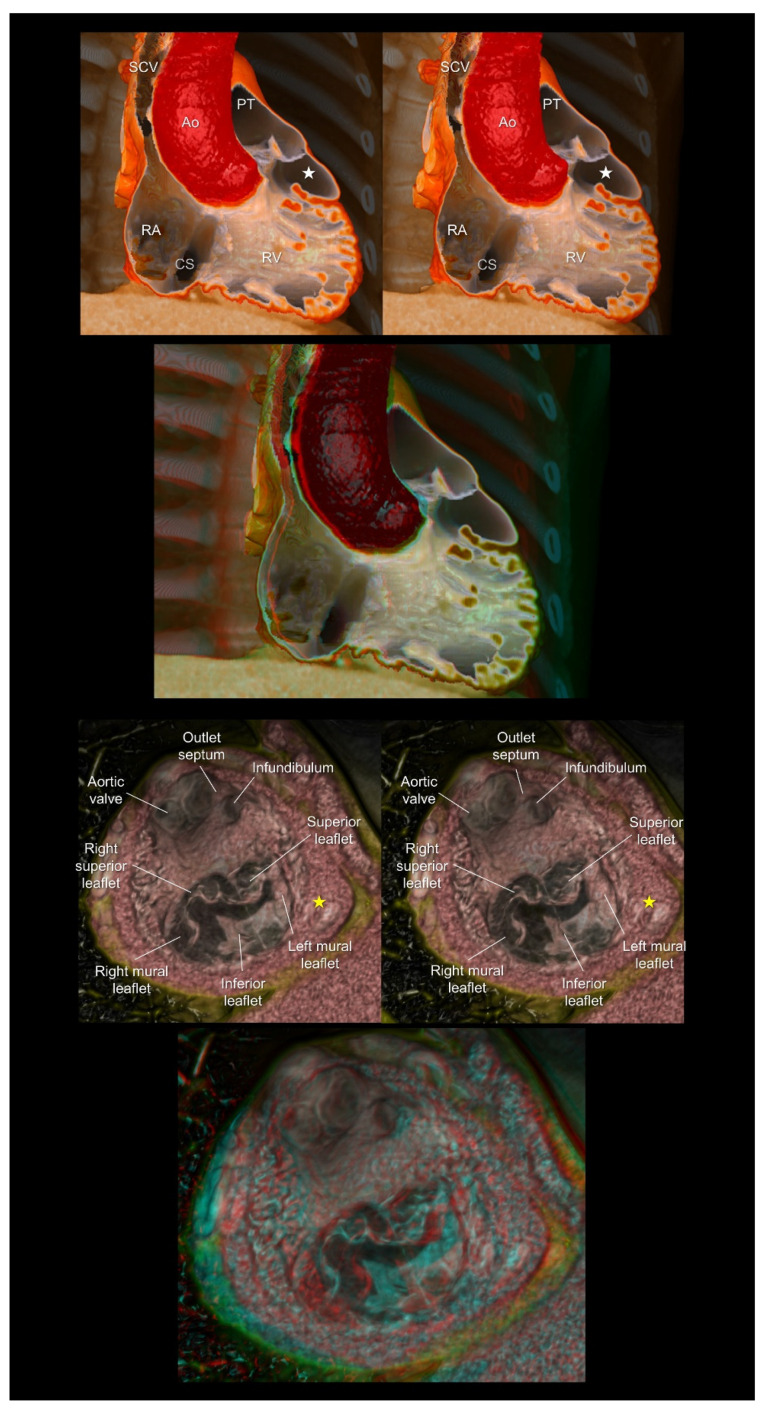
The (**upper panels**) show the double-chambered right ventricle with abnormally developed septoparietal trabeculations creating a low-pressure outflow chamber (white stars) beneath the pulmonary valve. The (**lower panels**) show a complex cardiac malformation case with right isomerism, a common atrioventricular junction, an atrioventricular septal defect, the double-outlet right ventricles, and subpulmonary infundibular stenosis. The yellow stars indicate a solidified rudimentary chamber. The first and second panels are aligned for cross-eyed viewing. The third panel is the anaglyph. To see the anaglyph, anaglyphic glasses (red/cyan) are required. Ao, aorta; CS, coronary sinus; PT, pulmonary trunk; RA, right atrium; RV, right ventricle; SCV, superior caval vein.

Congenital heart disease is often the most challenging feature for cardiologists in understanding individual three-dimensional anomalies. Therefore, a three-dimensional approach is essential [9,33] to share the complex anatomy and discuss the optimal therapeutic approach [10,11,34]. The more complicated the features of the involved heart, the more precise three-dimensional recognition is required, which provides a huge arena for stereograms in this field [16]. Cardiac magnetic resonance imaging is the gold standard for functional and morphological cardiac assessments [35,36,37]. However, it can provide insufficient spatial resolution for detailed anatomical analyses. With rapid technical improvements, including a significant reduction in radiation exposure and improved temporal resolution, the higher spatial resolution provided by cardiac computed tomography can work as a complementary tool to cardiac magnetic resonance imaging and three-dimensional echocardiography for the periprocedural assessment of the highly complicated morphology of congenital heart disease (Figure 7 and Figure 8) [38].

### 4.7. Cardiac Mass

**Figure 9:**
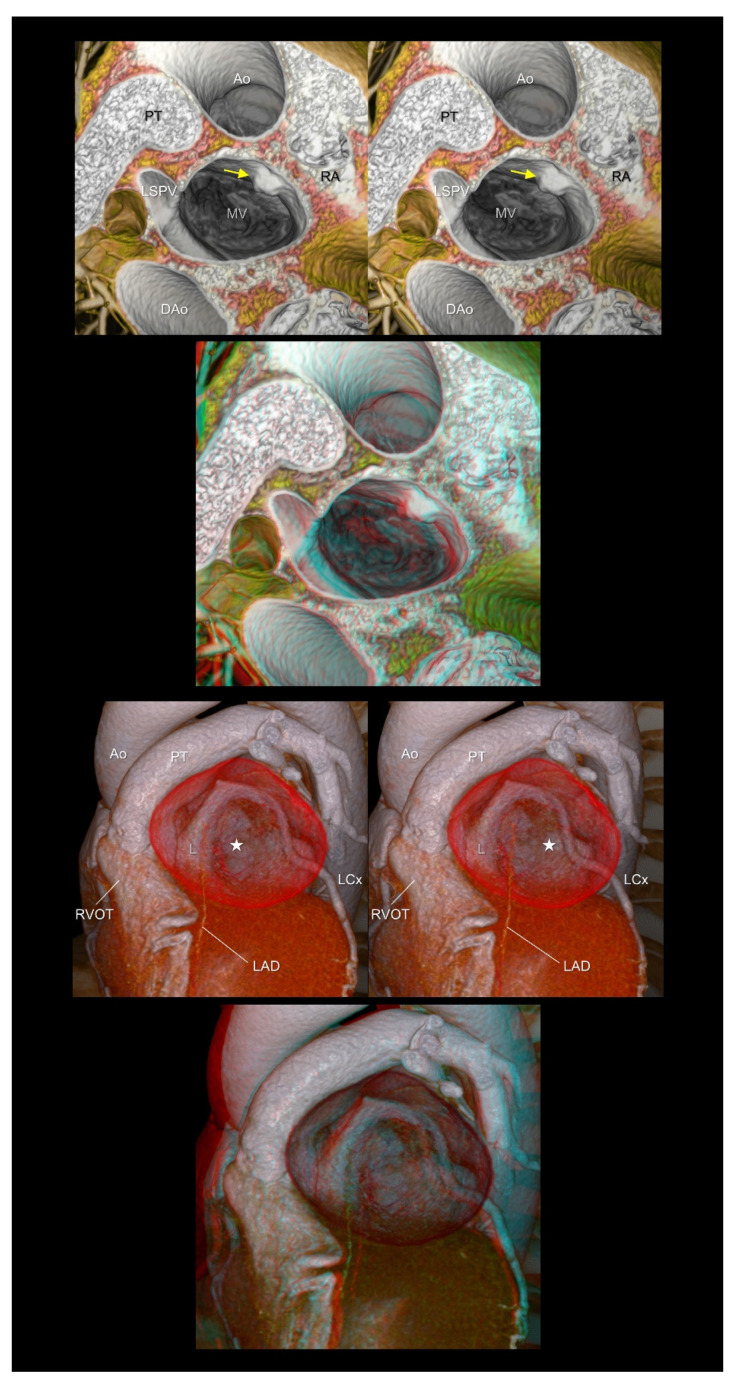
The (**upper panels**) show left atrial myxoma (yellow arrows). The (**lower panels**) show a giant coronary arterial aneurysm in the proximal left descending artery (white stars). The aneurysm is compressing or displacing the surrounding cardiac structures, including the right ventricular outflow tract, pulmonary root and trunk, left atrial appendage, and left circumflex artery. The first and second panels are aligned for cross-eyed viewing. The third panel is the anaglyph. To see the anaglyph, anaglyphic glasses (red/cyan) are required. Ao, ascending aorta; DAo, descending aorta; L, left coronary aortic sinus; LAD, left anterior descending artery; LCx, left circumflex artery; LSPV, left superior pulmonary vein; MV, mitral valve; PT, pulmonary trunk; RA, right atrium; RVOT, right ventricular outflow tract.

Clarifying the three-dimensional location of the intra- or extracardiac mass and its relationship with the surrounding structures is fundamental for estimating its potential impact on the cardiac structure and function [39]. Furthermore, in principle, a large or mobile intracardiac mass is subjected to potential surgical biopsy/resection, regardless of whether it is a tumor, thrombus, or vegetation. Therefore, the precise recognition of the three-dimensional anatomy influences the surgical strategy. In this regard, stereopsis helps in the understanding of the topographical relationship between the mass and the surrounding structural anatomy of the heart (Figure 9).

### 4.8. Virtual Procedural Simulation

**Figure 10:**
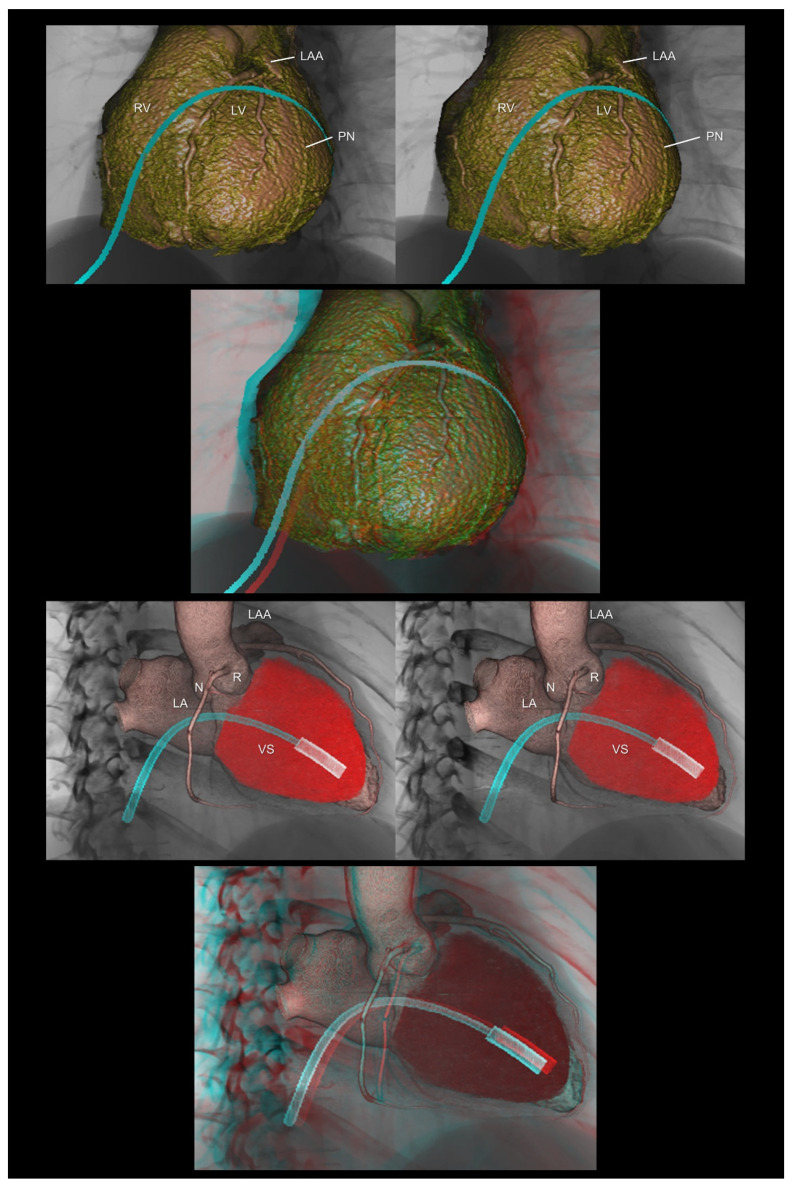
The (**upper panels**) demonstrate virtual epicardial ablation using the anterior approach via a subxiphoid access. The (**lower panels**) show a virtual delivery system of the leadless pacemaker. The first and second panels are aligned for cross-eyed viewing. The third panel is the anaglyph. To see the anaglyph, anaglyphic glasses (red/cyan) are required. LA, left atrium; LAA, left atrial appendage; LV, left ventricle; N, noncoronary aortic sinus; R, right coronary aortic sinus; RV, right ventricle; PN, phrenic nerve; VS, ventricular septum.

With the application of a specific interactive function installed in a workstation that is similar to computer-aided design, a three-dimensional virtual simulation of the invasive procedure is currently feasible [4,40]. Such digital data retaining three-dimensional coordinates can also be used for custom-device development with or without the assistance of three-dimensional printing. In this setting, a stereogram was used to assess the feasibility of the virtually reconstructed device (Figure 10).

## 5. Discussion

In 1838, Wheatstone published the concept of stereopsis and created the first stereoscope [12]. However, after 184 years, his discovery seems to be underutilized in clinical settings, despite the significant advancements in cardiac imaging examinations in the 21st century [1,2,3,4,5,41]. Despite the lack of depth perception, volume-rendered images displayed on the two-dimensional surface are deemed to be “three-dimensional” images in clinical settings. In this review, we revisited stereopsis as a simple, effective, and affordable way to obtain real depth perception.

The creation of a stereoscopic image requires the reconstruction of high-quality volume-rendered images [3], making the acquisition of high-quality raw image datasets fundamental. Therefore, in the setting of cardiac magnetic resonance imaging or computed tomography, communication and collaboration among cardiologists, radiologists, and radiologic technologists are vital to ensure appropriate imaging with justifiable use of radiation and contrast. This requires a customized approach, making preprocedural planning before imaging crucial. Furthermore, as we cannot reconstruct what we cannot see and/or what we do not know, a profound knowledge of the basic cardiac anatomy is essential. Otherwise, a stereogram generated based on anatomically inaccurate reconstructed images may lead to incorrect clinical decisions.

The preparation of stereograms is straightforward. Stereopsis can be readily achieved by simply displaying two volume-rendered images reconstructed with different rotation angles (10–15°) or anaglyphs with inexpensive anaglyphic glasses. Using a commercially available workstation, it is easy to create rotationally paired images. Generally, it takes less than one hour from the acquisition of the computed tomographic data to the image reconstruction and generation of stereograms.

Although high-quality volume-rendered images can be reconstructed using clinically obtained datasets [4], including computed tomography, magnetic resonance imaging, and three-dimensional echocardiography, a single-image display on a two-dimensional monitor cannot provide depth perception. As the three-dimensional datasets involve depth information innately, trying to generate stereograms can be deemed as an attempt to fully utilize patient data. These results are promising in terms of achieving dramatic depth perception, as shown in the present figures. If these stereograms are readily accessible via a picture-archiving communication system and anaglyphic glasses prepared in the ward or conference room, the utility of stereograms would expand broadly. This review could trigger the interest of clinicians to revisit stereograms independently. Moreover, the educational utility of volume-rendered images over two-dimensional images remains controversial [42]. Therefore, further investigation is necessary to evaluate the educational and clinical utilities [42] of real three-dimensional methodologies to obtain depth perception, with their cost-effectiveness and comparison among each other. In this regard, a readily accessible stereogram can function as an initial access to a real three-dimensional field.

Once high-quality volume-rendered images are obtained, three-dimensional datasets can be readily applied to stereograms, three-dimensional printing, three-dimensional projectors/monitors, and virtual reality applications to obtain depth perception. Each application has its own advantages and disadvantages in terms of user-friendliness, cost performance, and clinical relevance. Specifically, only three-dimensional printing can provide real three-dimensional feature with realistic textures of the reconstructed structures. Thus, three-dimensional printing is useful for surgical planning and simulation of the procedure [33,34]. On the other hand, if the heart is exclusively printed out, as is generally the case, it automatically loses the three-dimensional relationships with surrounding structures. This is a similar problem in the setting of real cardiac dissection. In this regard, it is of additional value that virtual three-dimensional applications, as shown in the present figures reconstructed from living hearts, can retain the physiological relationships with adjacent structures. This is feasible because the heart does not need to be “removed” from the thorax to create these images.

## 6. Conclusions

Representative stereograms have been obtained in multiple clinical settings in the field of cardiology. Before the two-dimensional appreciation of complicated three-dimensional structures, a direct three-dimensional appreciation should be achieved. Currently, with the development of three-dimensional imaging, this is readily feasible in clinical settings. Therefore, in the next stage, a real three-dimensional appreciation with depth perception should be attempted. Conventional stereopsis is an easy, convenient, and affordable method that does not require any special equipment, except for anaglyphic glasses. This technique can be immediately introduced into daily clinical practice and medical education.

## Data Availability

Not applicable.
